# Endobronchial Radiofrequency Ablation for pulmonary nodules with Radial-Ebus and Navigation: Pros and Cons

**DOI:** 10.7150/jca.84894

**Published:** 2023-05-21

**Authors:** Paul Zarogoulidis, Wolfgang Hohenforst-Schmidt, Wei Chen, Konstantinos Porpodis, Christoforos Kosmidis, Athanasios Kotsakis, Eleni-Isidora Perdikouri, Christos Tolis, Aris Ioannidis, Konstantinos Sapalidis, Dimitris Matthaios, Dimitrios Giannakidis, Panagoula Oikonomou, Christina Nikolaou, Vasilis Papadopoulos, Chrysanthi Sardeli, Charalampos Charalampidis, Savvas Petanidis, Chong Bai, Haidong Huang

**Affiliations:** 1 Pulmonary Department, General Clinic Euromedica, Thessaloniki, Greece.; 2 Sana Clinic Group Franken, Department of Cardiology / Pulmonology / Intensive Care / Nephrology, ''Hof'' Clinics, University of Erlangen, Hof, Germany.; 3 Department of Respiratory and Critical Care Medicine, The Affiliated Huaian No.1 People's Hospital of Nanjing Medical University, Huai'an 223300, China.; 4 Pulmonary Department, ``G. Papanikolaou`` General Hospital, Aristotle University of Thessaloniki, Thessaloniki, Greece.; 5 3 rd Surgery Department, AHEPA University Hospital, Aristotle University of Thessaloniki, Thessaloniki, Greece.; 6 Oncology Department, University General Hospital of Larissa, Larissa, Greece.; 7 Oncology Department, General Hospital of Volos, Greece.; 8 Private Oncology Clinic, Oncoderm, Ioannina, Greece.; 9 Surgery Department, Genesis Private Clinic, Thessaloniki, Greece.; 10 Oncology Department, General Hospital of Rhodes, Rhodes, Greece.; 11 1st Department of Surgery, Attica General Hospital "Sismanogleio - Amalia Fleming", Athens, Greece.; 12 Second Department of Surgery, University Hospital of Alexandroupolis, Medical School, Democritus University of Thrace, Alexandroupolis, Greece.; 13 Oncology Department, University General Hospital of Larissa, Larissa, Greece.; 14 Department of Pharmacology & Clinical Pharmacology, School of Medicine, Aristotle University of Thessaloniki, Thessaloniki, Greece.; 15 Department of Anatomy, University of Cyprus Medical School, Cyprus, Greece; 16 Department of Medicine, Laboratory of Medical Biology and Genetics, Aristotle University of Thessaloniki, Thessaloniki, Greece.; 17 Department of Respiratory & Critical Care Medicine, Changhai Hospital, the Second Military Medical University, Shanghai, P. R. China.

**Keywords:** bronchoscopy, rapid on-site evaluation, ROSE, radiofrequency ablation, radial-ebus, C-arm, lung cancer.

## Abstract

**Introduction:** Pulmonary nodules are common in the everyday clinical practice. There is always a diagnostic issue with this imaging finding. Based on the size we can use a variety of imaging and diagnostic techniques. Moreover; in the case of primary lung cancer or metastasis we can use radiofrequency ablation endobronchially.

**Patients and Methods:** We used the radial-endobronchial ultrasound with C-arm and Archemedes, Bronchus electromagnetic navigation in order to acquire biopsy sample and we also used rapid on-site evaluation as a rapid diagnosis for pulmonary nodules. After rapid diagnosis we used the radiofrequency ablation catheter in order to ablate central pulmonary nodules.

**Results:** Both techniques provide efficient navigation, however, with the Bronchus system less time is needed. The new radiofrequency ablation catheter provides efficient results in central lesions with low watts ≤40.

**Conclusion:** We provided in our research a protocol to diagnose and treat such lesions. Future larger studies will provide more data on this subject.

## 1. Introduction

Lung cancer is still diagnosed at advanced stage. We are currently trying to encourage all smokers, ex-smokers or people with exposure to toxic chemicals to have low dose computed tomography scans for screening.^1^ The main tools for lung cancer diagnosis are radial-endobronchial ultrasound, electromagnetic navigation, transthoracic biopsy under ultrasound or computed tomography scan.^2^-^6^ Currently we have an additional technique which we use in the diagnostic procedure where we can evaluate our sample firstly if we have cancer cells and secondly if we have enough sample for immunohistochemistry and next generation sequencing (NGS).^7^ Early stage lung cancer is usually found with pulmonary nodules on a computed tomography scans (CT). In the case of nodules ≥1cm in diameter we can perform positron emission tomography (PET-CT) as a method to acquire information regarding the metabolic rate and evaluate the lymphnode stations in the thorax and check for distant metastasis.^8^ In the case of pulmonary nodules less than 1cm the PET-CT usually cannot provide us with information regarding the metabolic rate due to the technical limitations of the method. However; we should not forget that even large nodules, large lymphnode stations can be false negative due to the low metabolic rate of the cancer disease.^9^ In the case of pulmonary nodules ≤1cm the concept until new diagnostic methods were developed was to re-evaluate the size of the lesion every 3 to six months with a computed tomography scan. If the size was increased then PET-CT would follow and based on the results biopsy of surgery was performed.^10^ In the case of inoperable pulmonary nodules due to low respiratory function or coronary heart disease radiofrequency ablation and microwave ablation were developed which were catheters inserted to the thorax under CT guidance. The main adverse effects were pneumothorax, hemothorax and of course they cannot be applied if the lesion is next to a large vessel. During the last decade the addition of radial-endobronchial ultrasound with C-arm, electromagnetic navigation and software programs have provided us with tools for diagnosis of small pulmonary nodules from 0.5mm to 3cm with central or peripheral location. The addition of rapid on-site evaluation has increased the diagnostic rate of these procedures. Furthermore; in the last five years a new equipment for endobronchial radiofrequency ablation was introduced. Again, the main issues are the navigation to the target site and that it can be used for central and less for peripheral mass. In our current study we evaluate in a long term the efficiency of an endobronchial radiofrequency catheter for pulmonary nodules up to 3cm.

## 2. Patients and Methods

### 2.1 Navigation to pulmonary nodules

**Figure 1:**
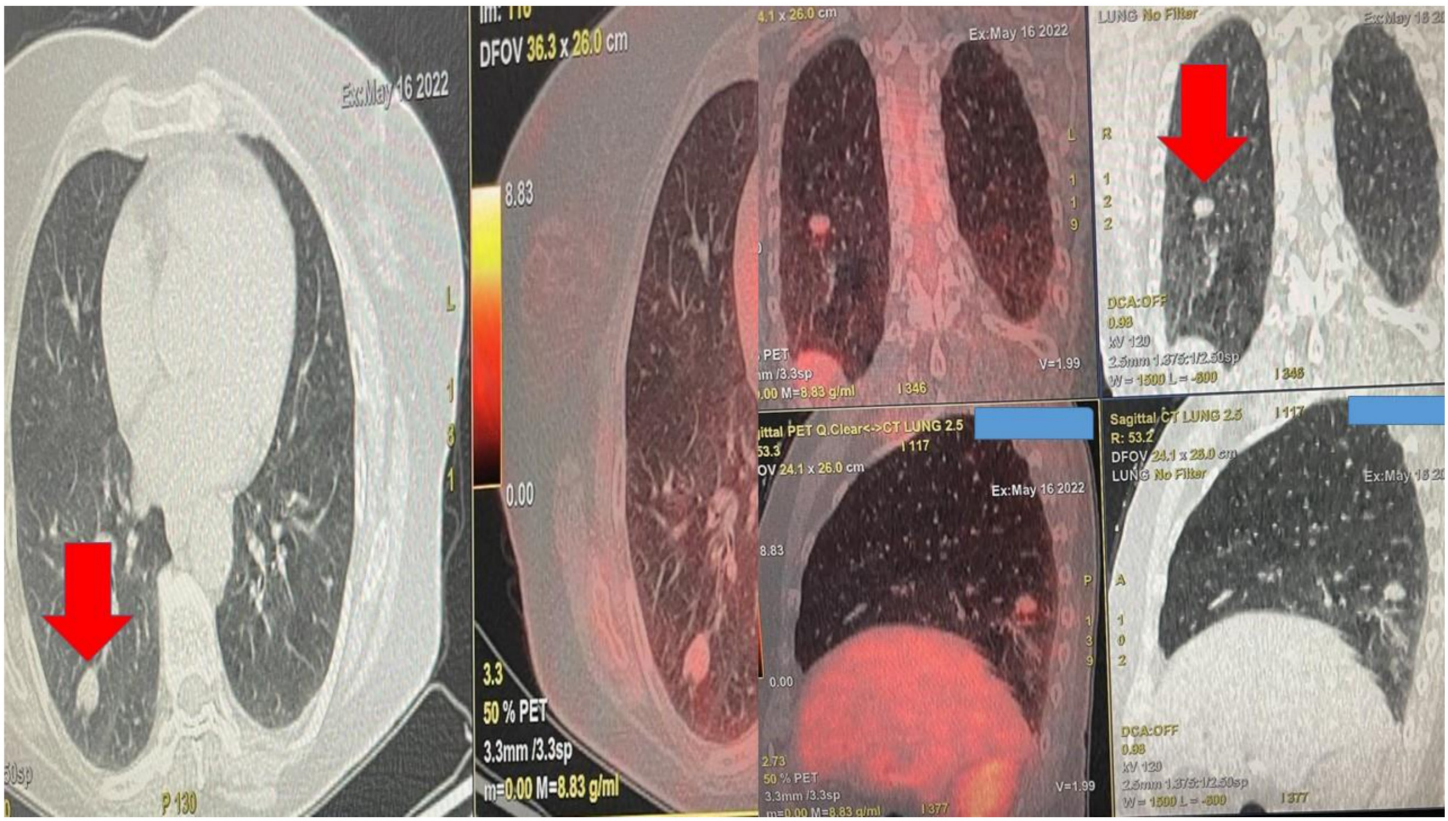
Demonstrates the positron emission tomography findings upon diagnosis red arrow indicates the pulmonary nodule.

**Figure 2:**
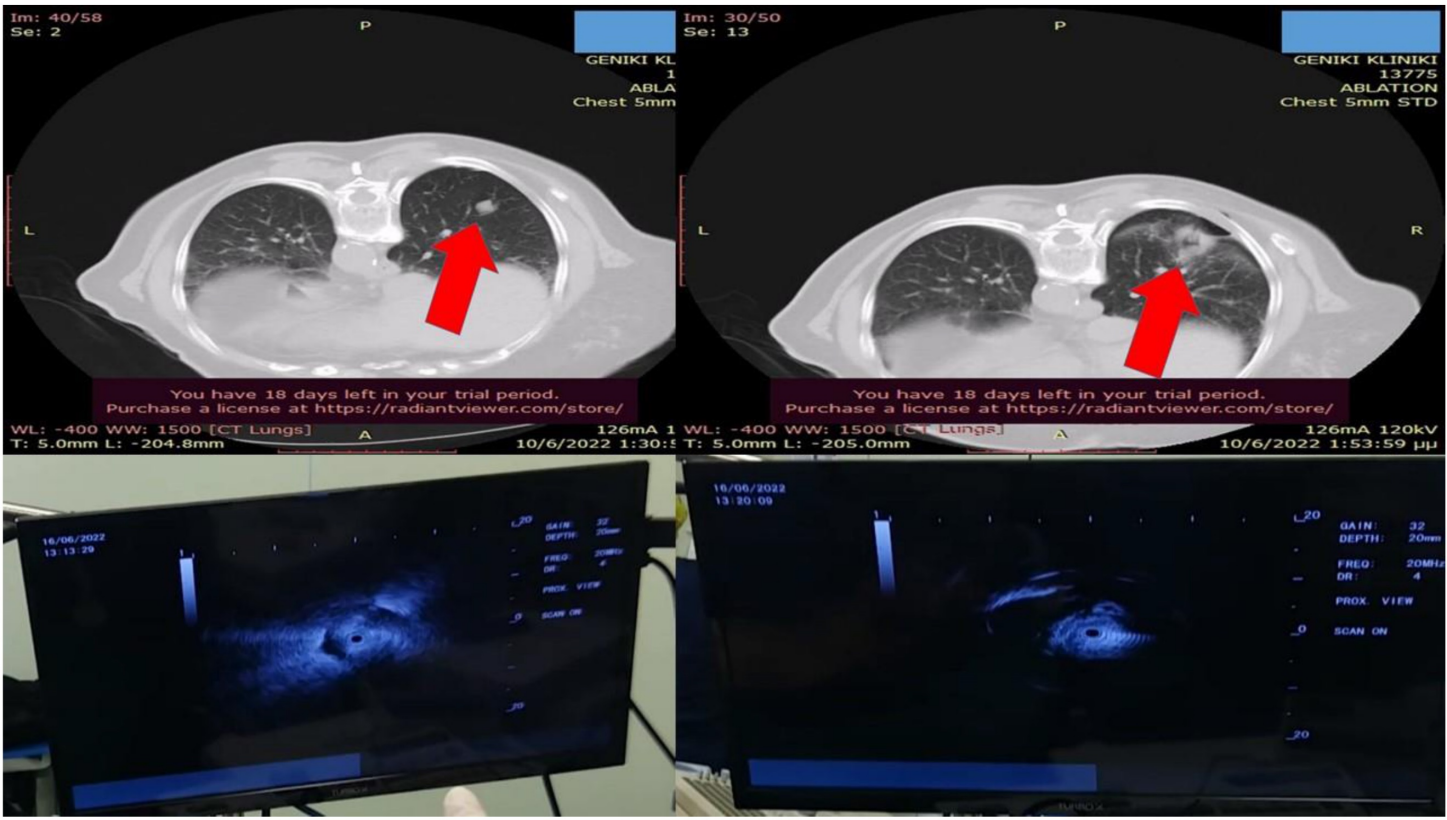
Left upper row red arrow indicates the lesion before ablation, left lower row demonstrates the lesion sign with radial ebus, right upper row red arrow indicates the area after 2 sessions of thermal effect with radiofrequency ablation, right lower row demonstrates the lesion sign after the ablation.

**Figure 3:**
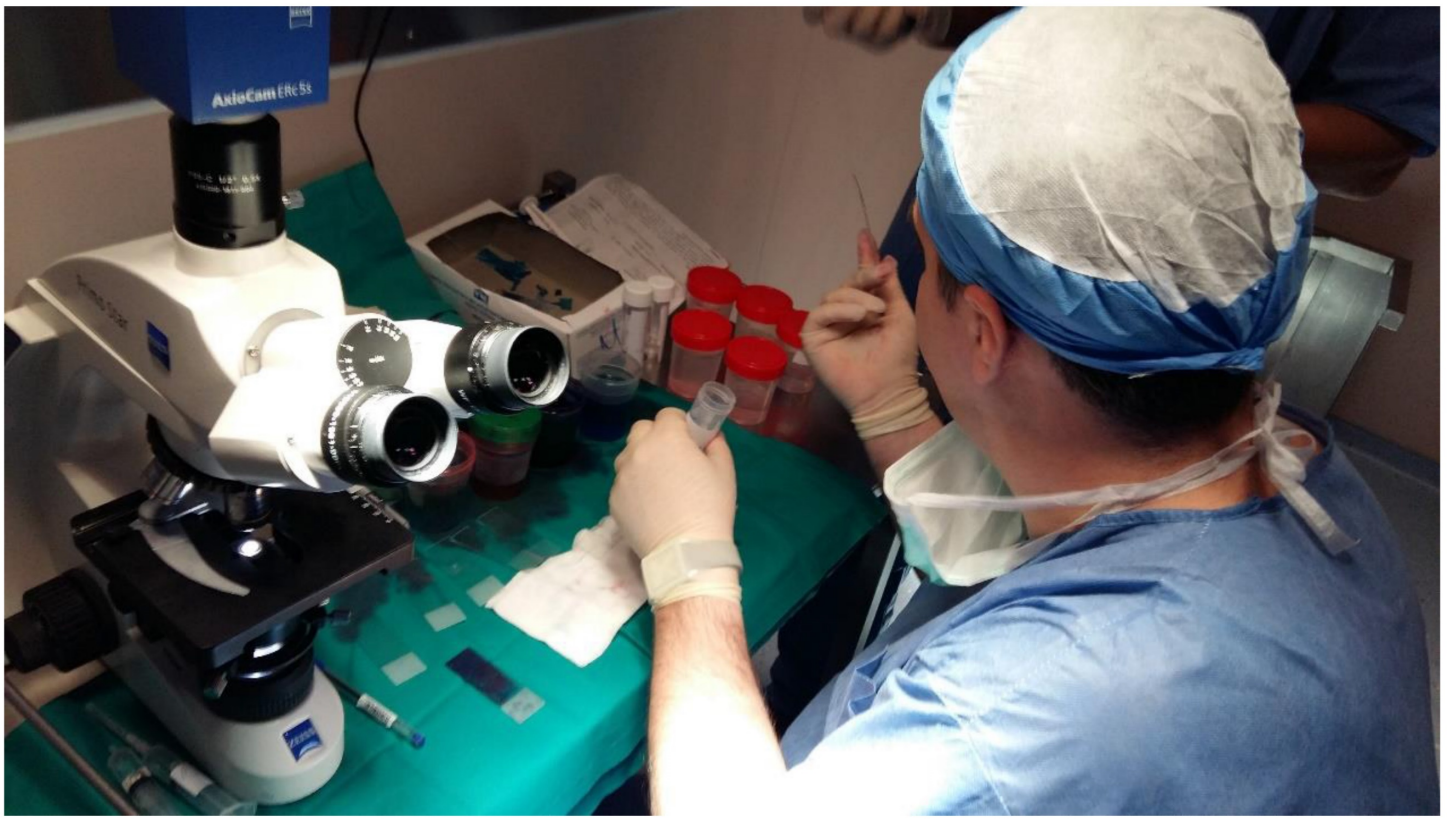
A pulmonary physician takes out the sample from the fine needle aspiration biopsy and prepares the specimen for evaluation under the microscope.

**Figure 4:**
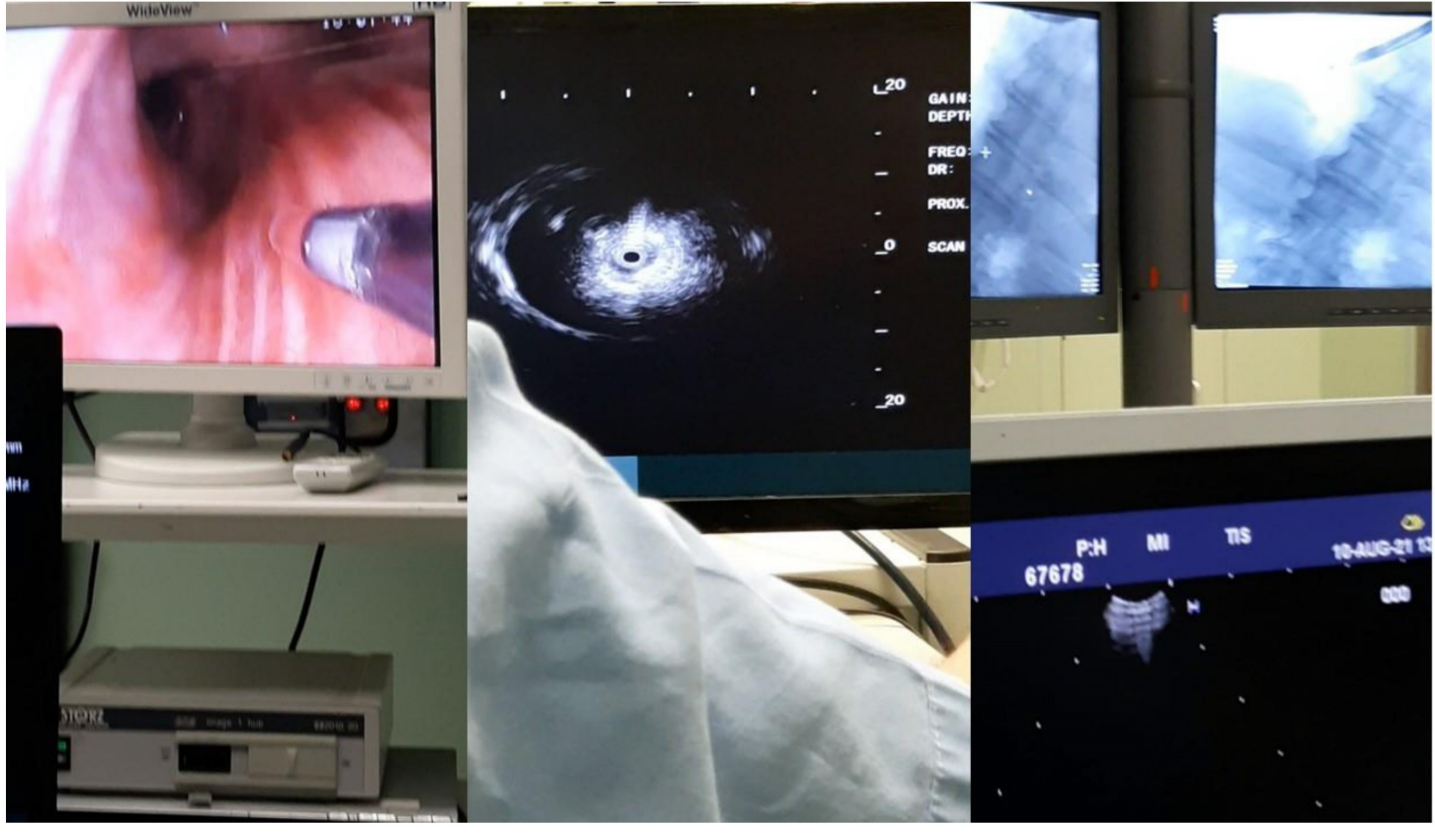
From left to the right the radial endobronchial ultrasound procedure under C-ARM fluoroscopy.

**Figure 5:**
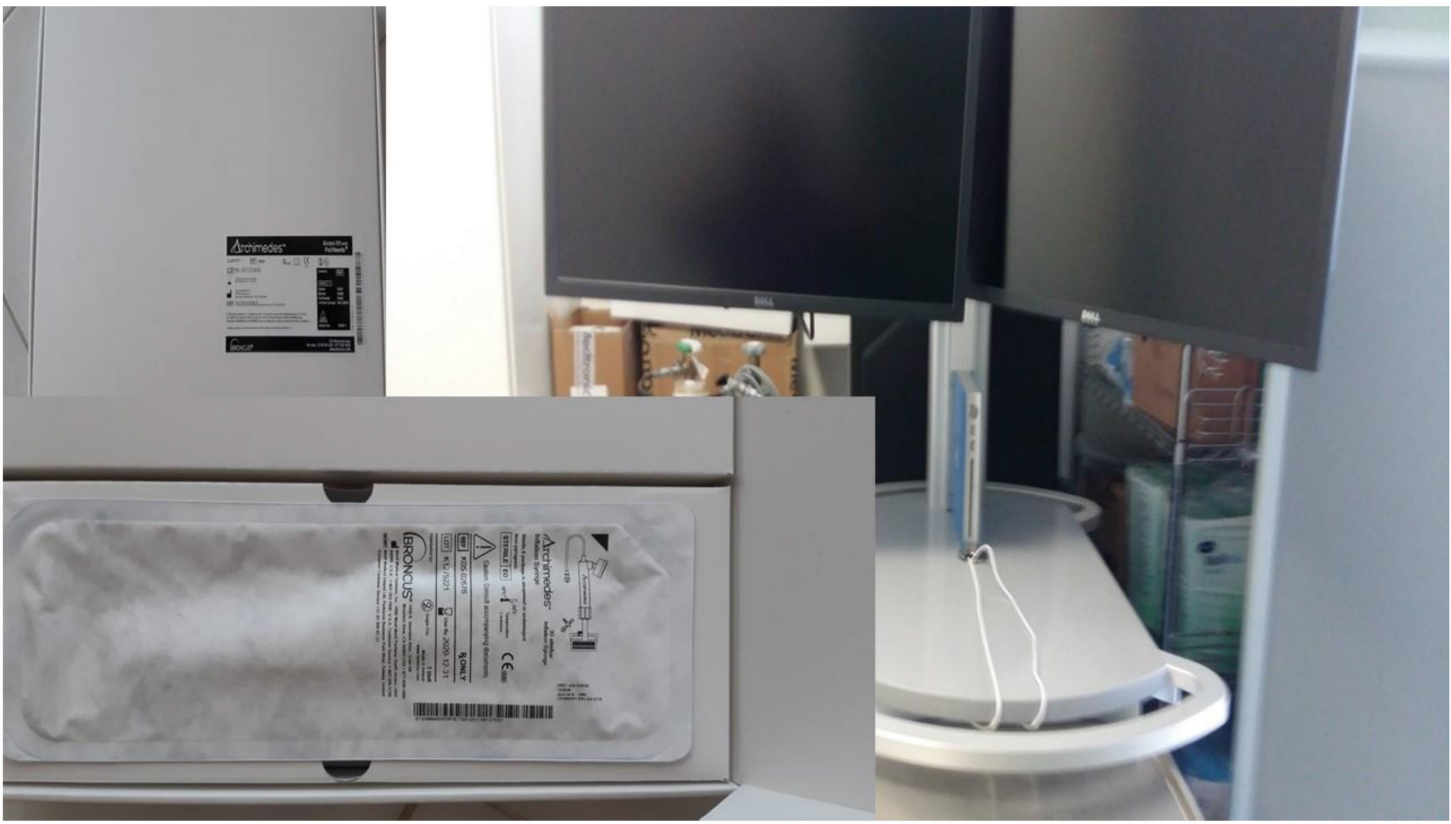
Bronchus system.

Three hundred patients were screened in our outpatient cabinet and in total one hundred and fifty-five patients were recruited with single or up to three pulmonary nodules in one lobe. The diameter of the nodules was between 1 and 2cm. Navigation to pulmonary nodules was performed with two systems a radial-endobronchial ultrasound Fuji plus a C-Arm (75 patients) and Archemedes, Bronchus electromagnetic navigation system (80 patients). The patients were divided into two groups and rapid on-site evaluation (ROSE) was performed in all patients. All patients recruited for this study had positive ROSE evaluated by a pulmonary physician and cytologist. Figure 1-5 The protocol was initiated in 2019 and terminated in July 2022.

Cone beam computed tomography (CBCT) is a well-established imaging modality with numerous proven applications across multiple clinical disciplines. More recently, CBCT has emerged as an important imaging tool for bronchoscopists, primarily used during transbronchial biopsy of peripheral pulmonary lesions (PPLS). For this application CBCT has proved useful in navigating devices to a target lesion, in confirming device tool-in-lesion, as well as during tissue acquisition. Intraprocedural 3D imaging with C-arm based CBCT was introduced in the early 2000s. CBCT images are obtained by rotating the C-arm approximately 200 degrees around the patient in a circular trajectory and acquiring a series of 2D X-ray projection images at specific angular intervals. Acquisition times range from 3-20 seconds and include up to 600 projection images, depending on the system type and imaging protocol, e.g., a 3-8 second acquisition duration is typical for body and cardiac applications. Projection images are then reconstructed, often on a separate workstation, using standard Feldkamp-based reconstruction algorithms, resulting in a 3D stack of axial images, analogous to conventional multi-slice CT (MSCT). With current detectors of 30 cm × 40 cm, CBCT acquisition covers a volume of 24×24×18.5 cm^3^ resulting in an isotropic volumetric dataset of 0.5 mm voxels in a 512×512 matrix. These reconstructed images can be reformatted into coronal, sagittal, and axial views of arbitrary thickness for reviewing.^11^, ^12^

Table 1-2. All patients were intubated or a rigid bronchoscope STORZ 12mm was used with jet-ventilation.

**Figure 7:**
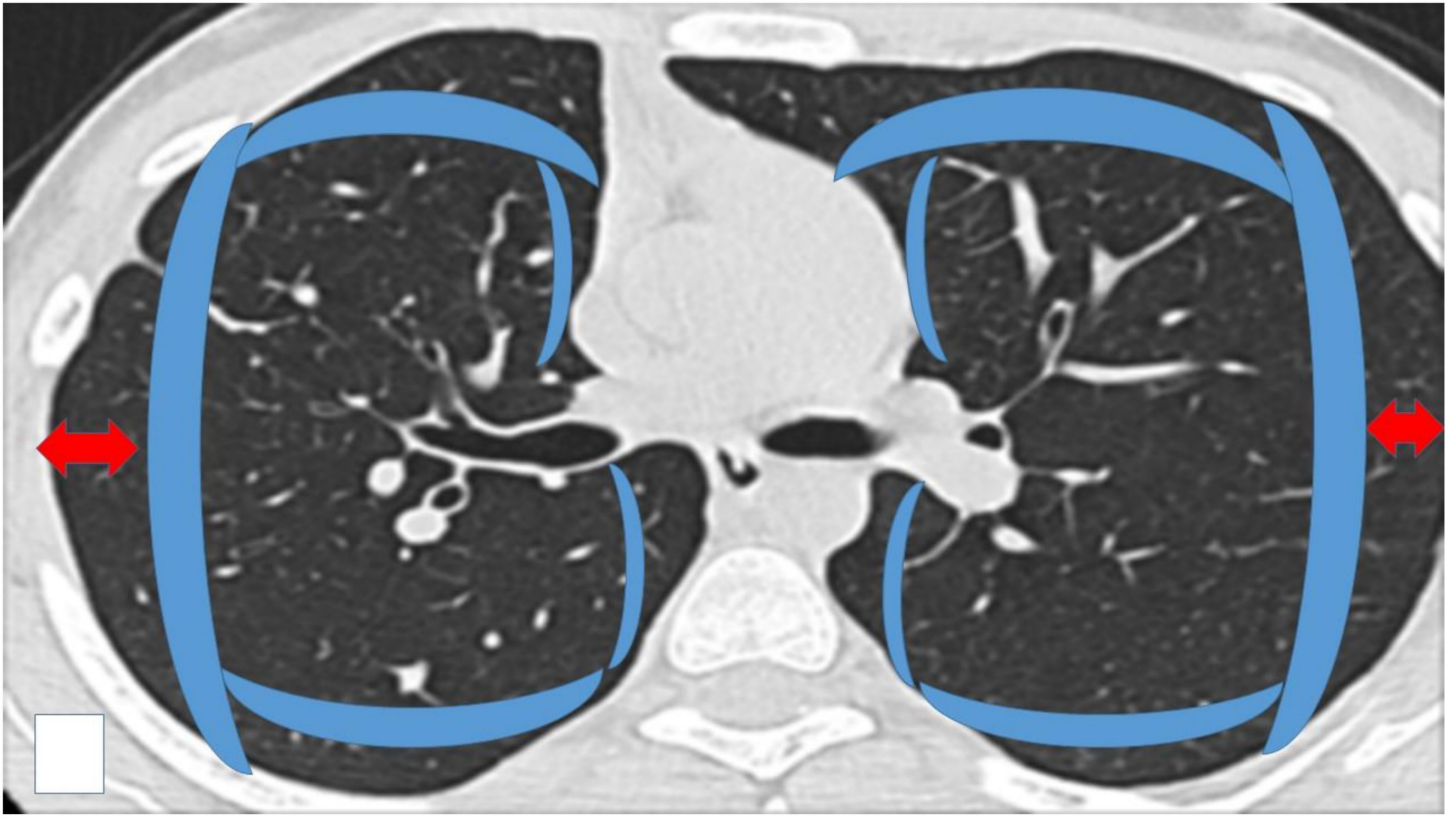
Schematic representation of the area considered as ``Central`` inside the blue boundaries and ``Peripheral`` (check red arrows). Central areas can be ablated easily by endobronchial catheter, however; there are cases where it is possible based on the local anatomy, surrounding vessels and size of the lesion.

All patients had previously to the endoscopy a positron emission tomography (PET-CT) performed and had the nodules evaluated as suspicious and of course there were no distant metastasis or metastasis in the mediastinum. All patients were informed and agreed that if a positive cytology was obtained then radiofrequency ablation would follow. In every patient the sample obtained was enough and immunohistochemistry was performed to obtain the exact histology diagnosis. All patients included had a sufficient respiratory capacity in order for the procedure to be performed. The one hundred and fifteen patients that were not included had evidence where the nodules had other primary disease such as prostate cancer, thyroid cancer, ovarian cancer and gastrointestinal cancer (45 Prostate cancer, 35 Gastrointestinal cancer, twenty Breast cancer and 15 Thyroid cancer). These patients were referred to our oncology board for evaluation. Moreover; we had sixty-five more patients diagnosed with a single pulmonary nodule or pulmonary nodules of ≤ 1cm. These patients had a negative PET-CT and were included in a close follow up protocol in our department with a three-month re-evaluation with computed tomography. Ten patients out of thirty-five were afterwards included in the protocol after minimum 9months because the size of the pulmonary nodules increased and the PET-CT was positive. These patients were not included in the study. All patients included had central lesions as presented in the figure 7 for technical reasons we could not approach peripheral nodules in thirty-five patients and therefore for these cases ablation under CT guidance is preferred. The data for these patients were not recorded in Tables 1 and 2. Mean time of hospitilisation for radial-ebus ablation 1.6 days, and for bronchus 1.4 days. Mean time of radial-ebus ROSE and ablation 45 minutes and for Bronchus-ROSE and ablation 40minutes.

### 2.2 Radiofrequency ablation system and methodology

We used a Covidien radiofrequency catheter 3cm length. We used 2-4 sessions of 20seconds each with 40 watts. The consistency of the nodule was evaluated before and after the procedure with the radial-endobronchial ultrasound in both groups of patients and a computed tomography was performed after each ablation procedure.

## 3. Results

**Figure 8:**
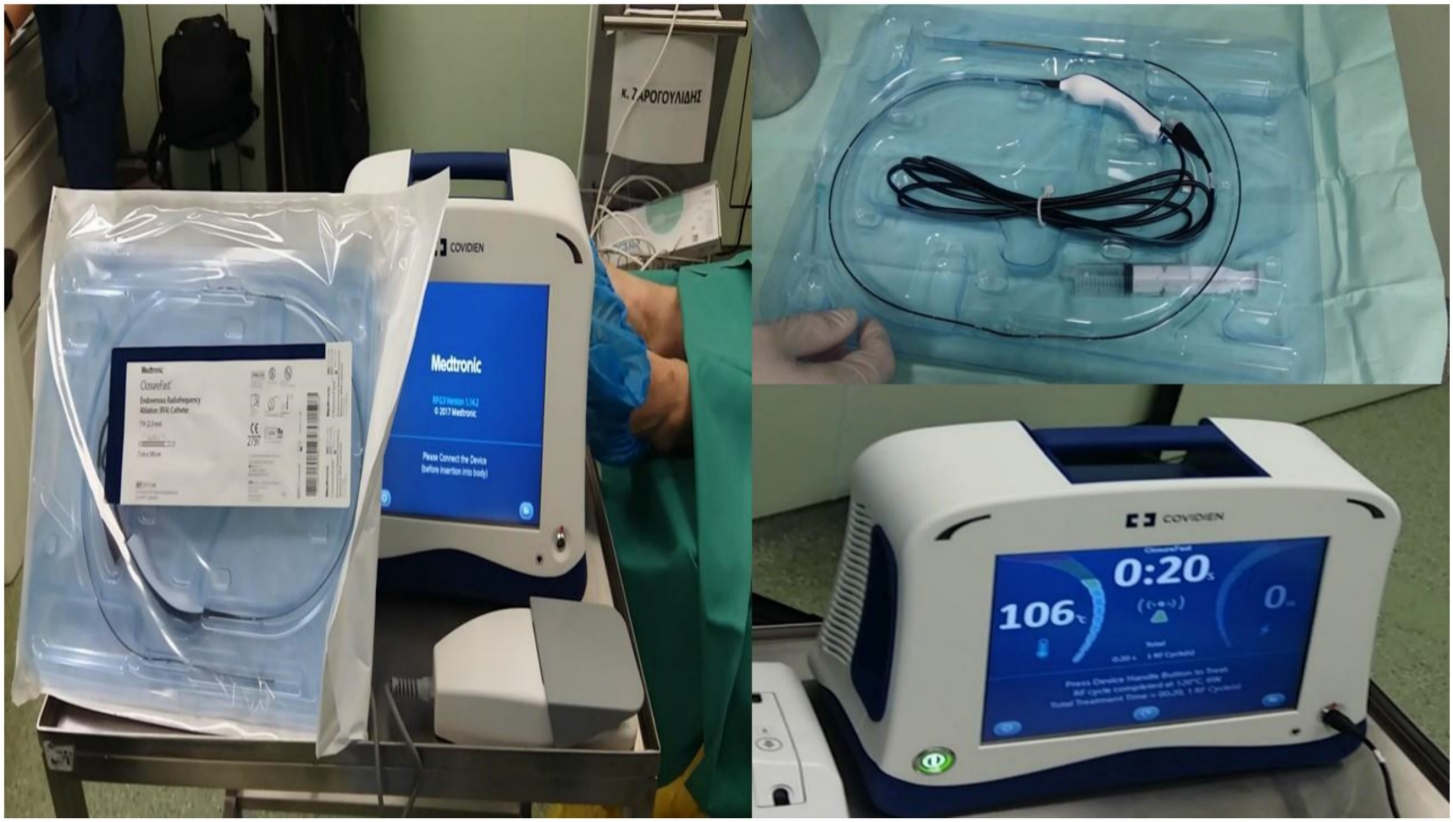
Covidien endovascular radiofrequency catheter.

**Figure 9:**
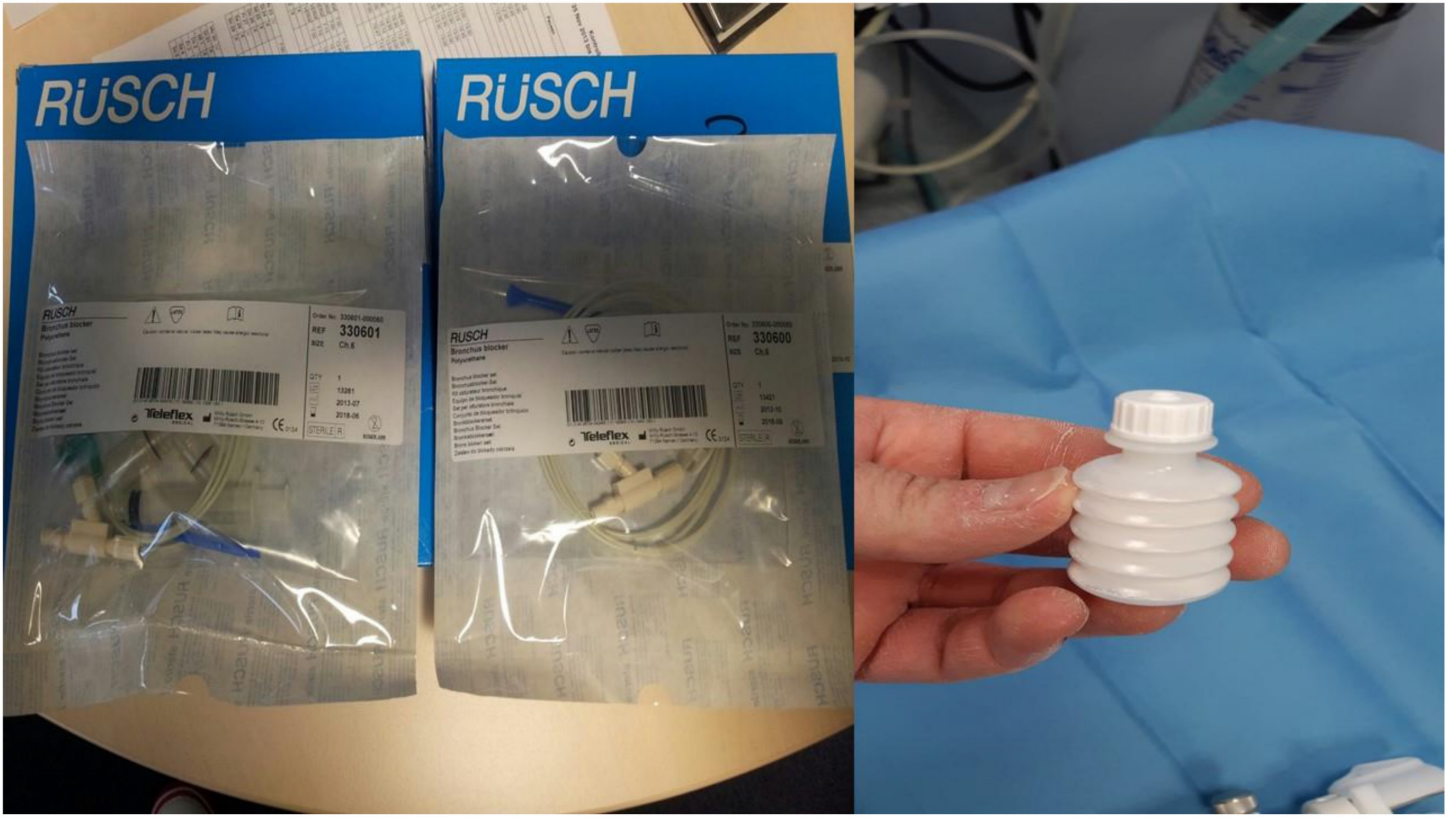
Ballon blocker and hemostatic powder.

Indeed, the results were extremely encouraging and verified with a long-term follow-up (one year). All patients included are one year after the procedure disease free. There were 80 cases where we had to change our approach to the lesions so that we could complete the ablation. Minor adverse effects were observed mostly being minor hemorrhage which was resolved with balloon blockers and special hemostatic powder sprayed in the region. Figure 8-9 Bronchoconstriction was also observed in 35 patients with chronic obstructive disease (COPD) which was resolved with the administration of intravenous cortisone, and aerosol bronchodilators and aerosol cortisone.

## 4. Discussion

In our study we included only lesions that were identified as positive with ROSE technique. Moreover; on purpose we did not choose patients with pulmonary nodules ≤1cm since we would definitely have adverse effects such as hemorrhage and possibly false negative results. We kept record of all the patients that were diagnosed with pulmonary nodules ≤1cm during a 2-year period. The twenty-five patients out of thirty-five we still have them under follow-up and we will perform endoscopic diagnostic procedures if the size will change. The rapid on-site evaluation was performed by pulmonary physicians with experience on this method and a second evaluation was also performed by a cytologist. In our study we did not evaluate the efficiency of radial-endobronchial ultrasound versus Archemedes, Bronchus system, however; we evaluated the time of each procedure and adverse effects. This comparison has been performed in previous studies.^13^-^15^ The cost of the procedure with the radial-ebus under fluoroscopy is the same as with the electromagnetic navigation, however; the cost of the equipment is 5 times more regarding the electromagnetic navigation. The main advantage of the electromagnetic navigation is the visualization of small and large vessels in real time and time to diagnosis is by 30% less. However; our findings might differ from other centers based on experience with the two techniques. There was no difference between the types of lung cancer regarding the efficiency of the ablation technique.

Data presented from previous studies provide evidence that radial-endobronchial ultrasound with the addition of C-Arm and whenever possible a navigation software is an efficient diagnostic tool even for small lesions of ≤1cm. This technique has even higher results when combined with rapid on-site evaluation, and it should always be combined with ROSE.^2^, ^4^, ^16^, ^17^ Electromagnetic navigation systems are already on the market in the last 10 years with very high diagnostic results, however; again rapid on-site evaluation should be applied to avoid unnecessary false negative results.^18^, ^19^ In our study both techniques were efficient with the addition of rapid on-site evaluation, with electromagnetic navigation being faster as a process. Rapid on-site evaluation is performed both by pulmonary physicians and cytologists, it is only a matter of experience. Several centers have applied this technique in their everyday clinical practice. In other centers catheters producing steam for the treatment of emphysema are being used for thermal ablation of small nodules.

## 5. Conclusion

In conclusion the electromagnetic navigation system cost is an issue, however; the cost per procedure is cost effective. Definitely the visualization of the surrounding vessels is an advantage and moreover; the time of each procedure is less. The radiofrequency ablation is a cost-effective equipment when compared to surgical procedures. Regarding peripheral lesions where the endobronchial system is unable to approach then we should check the distance from the catheter tip in order to evaluate the damage induced to the surrounding normal tissue and lesion. Based on the experience we should either choose to proceed or refer our patient for ablation under CT guidance. The major advantage of the radial-endobronchial ultrasound is the real time evaluation of the ablation procedure. The user compares the initial image of the radial-ultrasound with the image after the process. If necessary, the ablation can be continued for more time. However; the definite results or adverse effects are observed after a computed tomography scanned is performed right after the procedure.

## Figures and Tables

**Figure 6:**
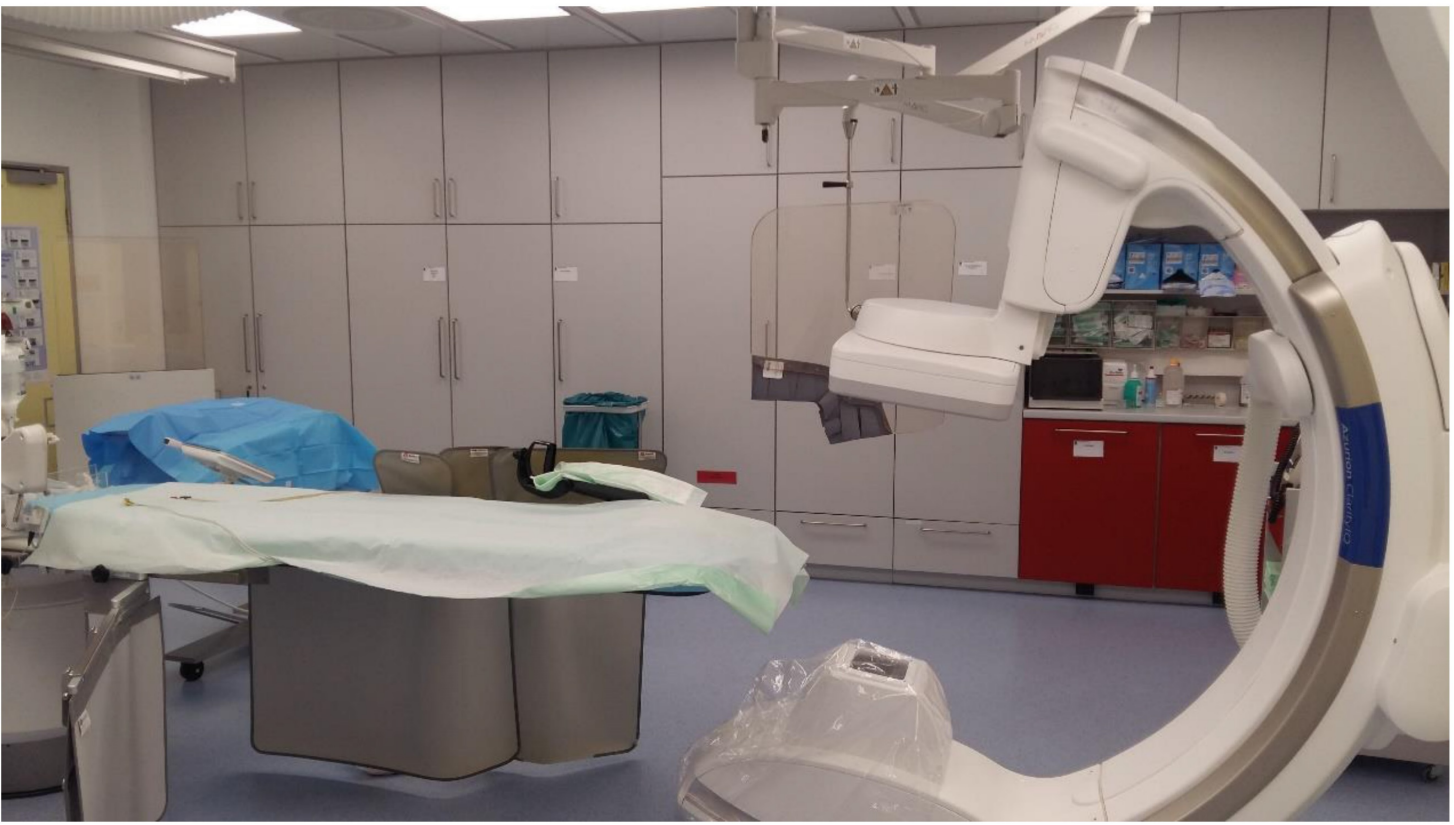
Dyna CT system Philips.

**Figure 10:**
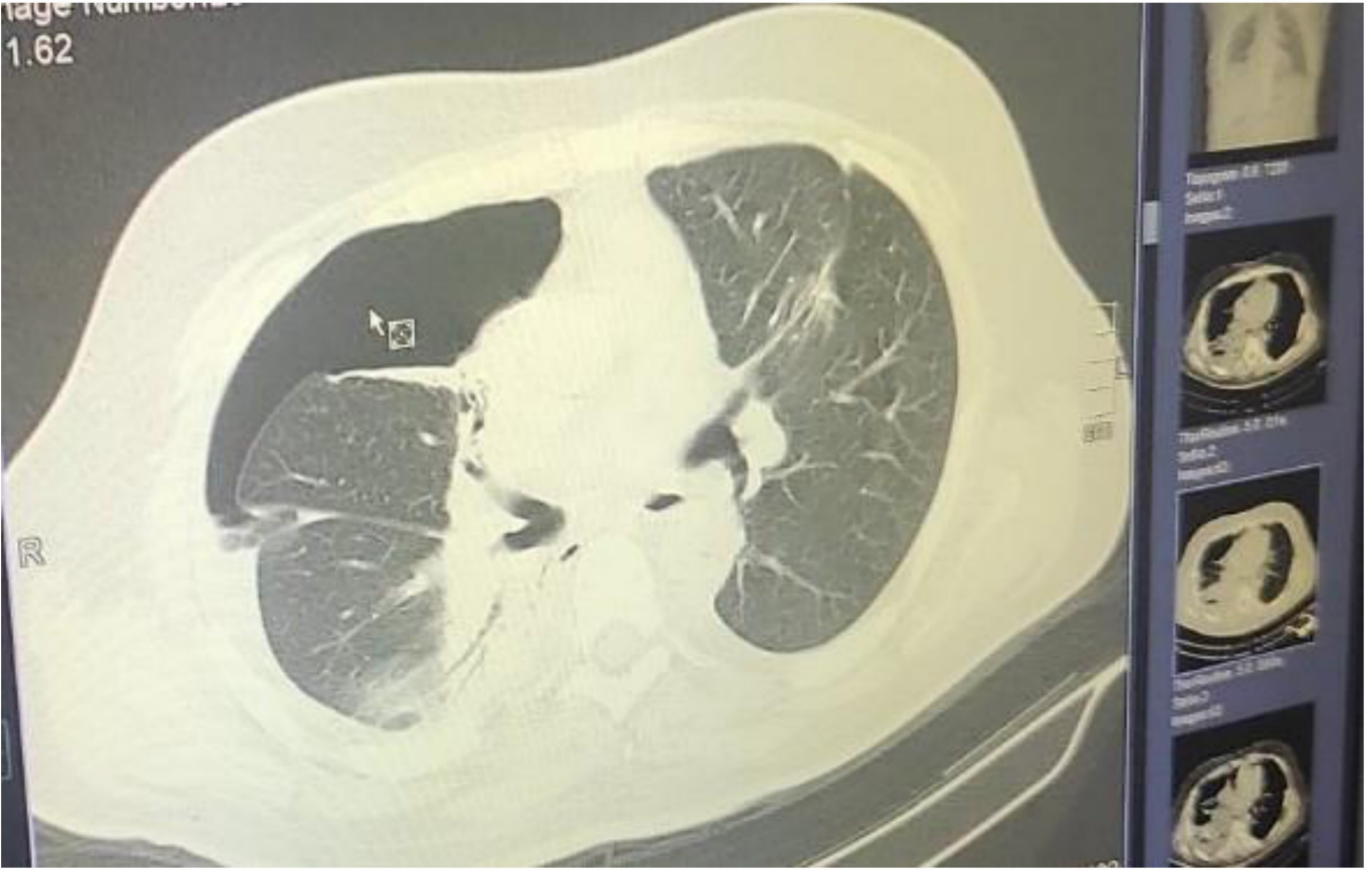
Pneumothorax after 2 days.

**Figure 11:**
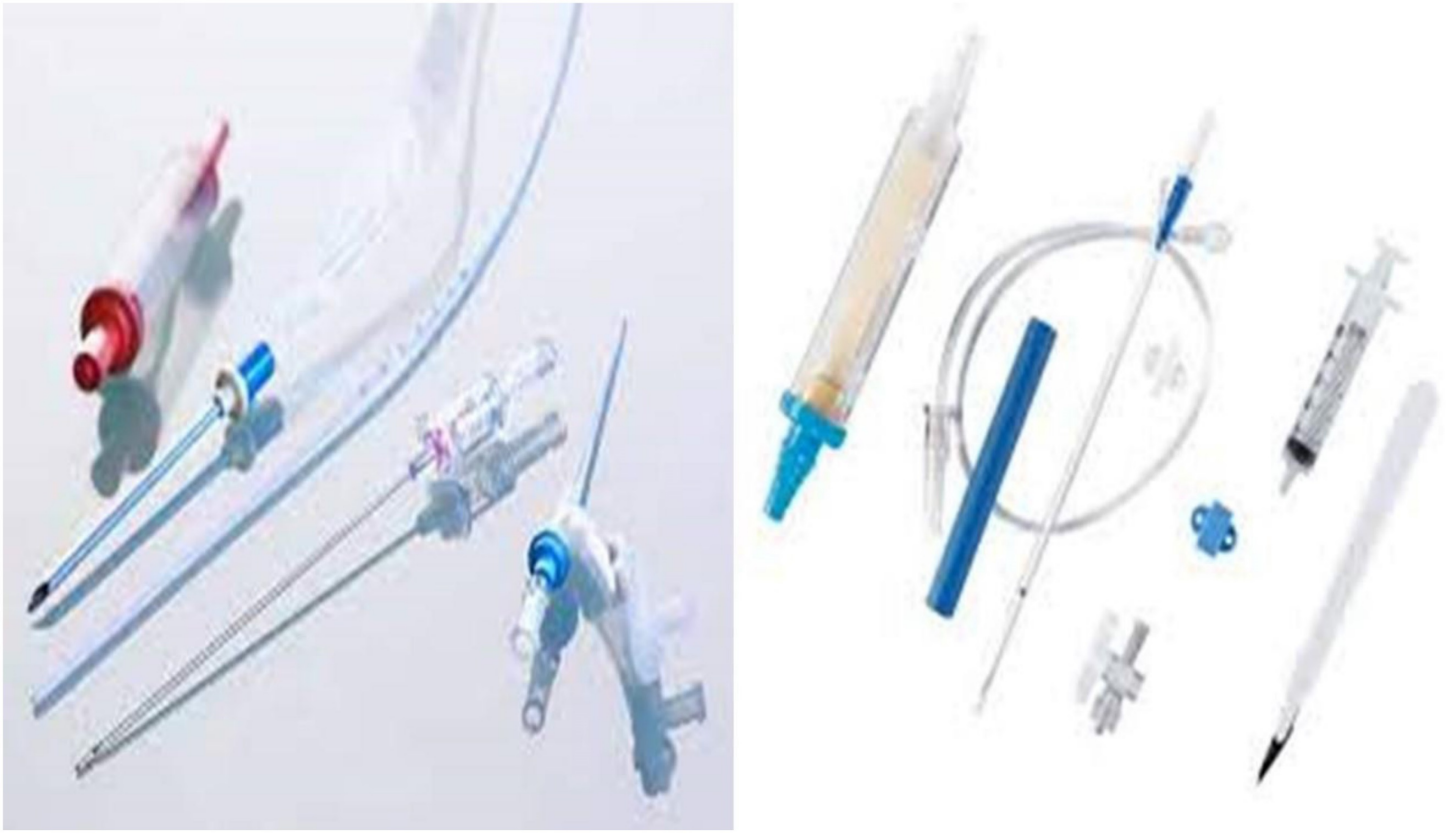
Two different types of pneumocather drainage systems used for pneumothorax.

**Figure 12:**
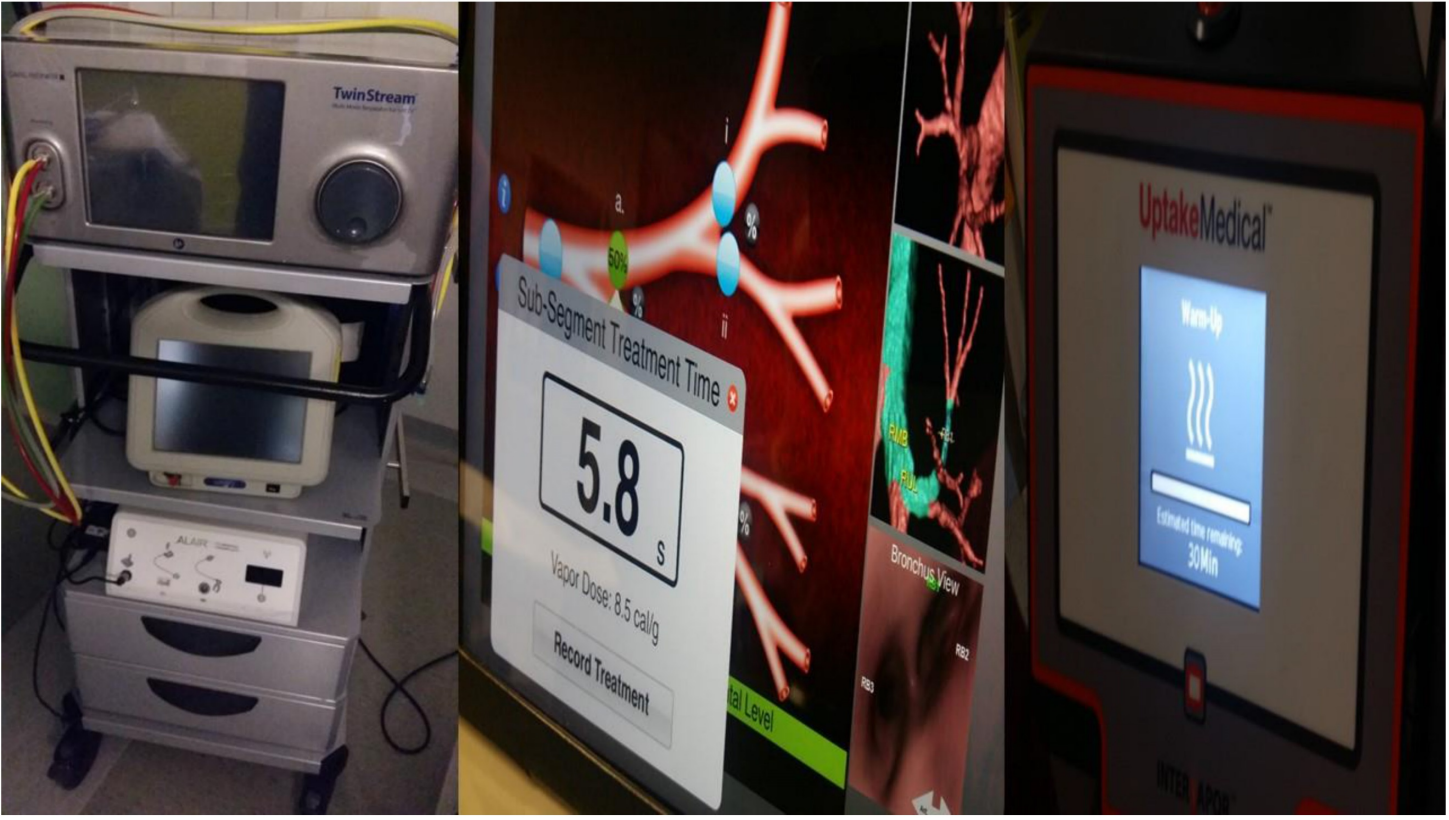
Thermal ablation system for emphysema and pulmonary nodules.

**Table 1:** Demographic data.

	RADIAL-EBUS		BRONCHUS	
SEX	40 MALE	35 FEMALE	45 MALE	35 FEMALE
NODULES PTS				
1	21	24	34	16
2	20	9	18	7
3	1	-	4	1
MEAN AGE OF PTS	65		63	
HISTOLOGY	43 ADENOCARCINOMA 32 SQUAMOUS		44 ADENOCARCINOMA 36 SQUAMOUS	

**Table 2:** Nodule size and time to diagnosis and ablation.

SIZE of nodules NUMBER PTS	RADIAL-EBUS		BRONCHUS	
1CM	40		55	
2 nodules 1-2CMS	29 18/1cm 7/2cm		25 10 /1cm 15/2cm	
3 nodules 1-2cms	1 1/1cm 1/2cm 1/2cm		5 Nodules mean Size 1.6cms	
